# Multi-ancestry Transcriptome-Wide Association Study (TWAS)-Informed Prioritization of Antipsychotic Metabolic Risk: Evaluation of GLP1R as a Shared Mechanistic Link

**DOI:** 10.7759/cureus.112759

**Published:** 2026-07-16

**Authors:** Ngo Cheung

**Affiliations:** 1 Psychiatry, Cheung Ngo Medical Limited, Hong Kong, HKG

**Keywords:** aggravating gene expression, antipsychotics, cardiovascular risk, compensatory gene expression, diabetes mellitus, drug-gene interaction, metabolic risk, pharmacogenomics, transcriptome-wide association study, twas

## Abstract

Antipsychotic-associated metabolic toxicity remains one of the most persistent clinical problems in psychopharmacology. Clozapine and olanzapine are especially effective for psychosis but carry high liability for weight gain, dyslipidemia, insulin resistance, and type 2 diabetes. Current monitoring recommendations recognize this risk, yet they remain largely uniform across patients and do not incorporate ancestry-specific genetic risk or mechanistic drug-gene information. We performed a transcriptome-wide association study-informed drug-gene prioritization analysis to examine whether approved antipsychotic target genes overlap with genes whose genetically predicted expression is associated with type 2 diabetes. The analysis used ancestry-specific type 2 diabetes transcriptome-wide association study (TWAS) results derived from a multi-ancestry genome-wide association study (GWAS) and approved antipsychotic drug-gene interactions from the Drug-Gene Interaction Database (DGIdb). For each drug, target genes were matched to TWAS genes across six metabolic tissues, and a weighted risk score was calculated as the sum of the absolute TWAS z-score multiplied by the drug-gene interaction score for significant targets. Follow-up analyses decomposed signals into targets with positive and negative TWAS directions, operationally interpreted as aggravating and compensatory, while also examining curated metabolic axis genes including GLP1R, GIPR, PPARG, and SLC2A4.

The analysis identified recurrent exploratory target-overlap signals for clozapine and olanzapine. Clozapine showed the most consistent cross-ancestry aggravating profile, with recurrent target overlap involving GLP1R and immune-related genes. Olanzapine showed strong mechanistic-axis overlap involving GLP1R, GIPR, and PPARG, although its simple TWAS directionality was often classified as compensatory. Trifluoperazine emerged as a notable candidate, with significant target enrichment in the European ancestry analysis and top ranking in the Hispanic analysis. Fluspirilene also met the combined enrichment false discovery rate threshold in the European ancestry analysis, although its clinical metabolic interpretation was less direct. Haloperidol decanoate showed a high burden driven partly by SLC2A4, but its directionality was frequently mixed or compensatory. These findings nominate the incretin axis as a plausible translational bridge between antipsychotic metabolic liability and existing interventions such as GLP-1 receptor agonists. They also identify a key methodological gap. Future models must incorporate the pharmacologic mode of action to distinguish receptor blockade from activation. Except for the enrichment-positive European ancestry findings for trifluoperazine and fluspirilene, the results are exploratory prioritization signals and do not establish drug-specific metabolic effects, causal mechanisms, or ancestry-specific treatment effects. Overall, this TWAS-informed analysis provides a hypothesis-generating framework for ancestry-aware metabolic monitoring and targeted validation studies.

## Introduction

Psychosis occurs in schizophrenia spectrum disorders and in several mood, substance-related, and medical conditions. For chronic psychotic disorders, long-term antipsychotic pharmacotherapy is often needed to control symptoms and reduce relapse. Antipsychotic drugs act predominantly through dopamine D2 receptor antagonism or partial agonism, while many agents also affect serotonergic, histaminergic, muscarinic, and adrenergic receptors. Differences in these pharmacologic profiles contribute to variation in both therapeutic effects and adverse-effect burden.

Metabolic adverse effects are a major limitation of antipsychotic treatment [1]. Weight gain, impaired glucose regulation, dyslipidemia, insulin resistance, and new-onset diabetes are common enough to influence drug selection, adherence, long-term morbidity, and mortality. The problem is most familiar for clozapine and olanzapine, which repeatedly rank among the antipsychotics with the greatest metabolic burden in clinical reviews and comparative meta-analyses. Quetiapine is generally associated with an intermediate average metabolic burden, whereas drugs such as aripiprazole and ziprasidone tend to show lower average liability [2-6]. Early consensus guidance from diabetes, endocrine, obesity, and psychiatric organizations recognized the need to monitor glucose, lipids, weight, and other metabolic parameters in people receiving antipsychotics [1]. Subsequent reviews and network meta-analyses have reinforced the same pattern. Clozapine and olanzapine are consistently among the highest-risk agents, while drugs such as aripiprazole and ziprasidone tend to show lower average metabolic liability [2-6].

Despite this clinical knowledge, the mechanisms linking antipsychotic exposure to diabetes remain incompletely resolved. Sedation, appetite stimulation, altered satiety, adipose dysfunction, insulin resistance, pancreatic effects, inflammatory activation, and receptor-level pharmacology have all been implicated. Yet clinical practice often still relies on broad risk categories rather than individualized risk prediction. Current monitoring guidance is therefore necessary but blunt. It helps identify metabolic deterioration after it begins, but it does less to explain why a given patient is vulnerable, why risk differs across drugs, or whether particular biological pathways could guide prevention. Guidelines from psychiatric and public health organizations recommend routine monitoring and management of cardiometabolic risk in severe mental illness, but they do not yet provide ancestry-informed or genetically anchored monitoring schedules [7-9].

This gap matters because type 2 diabetes is not a single-pathway disorder. It develops through multiple biological routes, including impaired insulin secretion, insulin resistance, adipose dysfunction, obesity-related mechanisms, liver and lipid metabolism, and vascular or inflammatory processes. Suzuki et al. [10] recently assembled a large multi-ancestry genome-wide association study (GWAS) of type 2 diabetes, including more than 2.5 million individuals and more than 428,000 cases, and identified 1,289 independent association signals mapping to 611 loci. Their analysis emphasized heterogeneity across ancestry groups and showed that type 2 diabetes-associated variants cluster into distinct cardiometabolic mechanisms. It also highlighted relevant regulatory tissues and cell types, including pancreatic islets, adipocytes, endothelial cells, enteroendocrine-related biology, and other metabolic contexts. This resource provides a useful background for asking whether drug targets intersect with genetically regulated expression programs associated with diabetes. The use of ancestry-specific data in the present study was intended to retain potential differences in the available GWAS and expression-prediction datasets; it was not intended to assign biological risk or treatment decisions to an individual on the basis of ancestry.

Transcriptome-wide association studies (TWASs) provide one route to address this question. PrediXcan and related TWAS approaches test associations between genetically regulated gene expression and disease or quantitative traits, using transcriptome prediction models trained in reference datasets [11]. Summary-based extensions such as S-PrediXcan allow gene-level association testing from GWAS summary statistics and support tissue-specific scans across multiple GTEx tissues [12]. These approaches do not prove causality by themselves, and they can be affected by linkage disequilibrium, reference panel choice, tissue context, and model performance. Still, they help move from variant-level association to gene-level hypotheses and provide a principled way to prioritize genes and pathways for follow-up.

The present analysis used ancestry-specific type 2 diabetes TWAS results derived from the Suzuki et al. GWAS [10] and asked a translational question: do approved antipsychotic target genes overlap with genes whose genetically predicted expression is associated with diabetes? The goal was not to estimate the clinical incidence of diabetes for each drug. Rather, the aim was to quantify genetic-expression proximity between antipsychotic target biology and diabetes-associated expression programs. The analysis was designed to recover known high-risk drugs as positive controls, identify ancestry-specific or less expected candidates, separate upregulated and downregulated diabetes-associated target hits, and examine metabolic-axis genes with direct translational relevance. Particular attention was given to clozapine, olanzapine, trifluoperazine, haloperidol decanoate, GLP1R, GIPR, PPARG, and SLC2A4 because these were the most informative signals across the primary and follow-up analyses. The analysis was intended to nominate candidate drug-target-diabetes expression overlaps for experimental and clinical validation, rather than to establish causal mechanisms, predict individual diabetes risk, or recommend treatment selection.

## Materials and methods

Approved antipsychotic drug targets were obtained from a Drug-Gene Interaction Database (DGIdb)-derived drug-gene interaction file. Drug names and gene symbols were standardized to uppercase, and only interactions annotated as approved were retained. Duplicate drug-gene pairs were collapsed by keeping the maximum interaction score. DGIdb was used because it integrates drug-gene interaction evidence from multiple resources and is commonly used for druggable gene and drug-target analyses [13-15]. The DGIdb interaction score was treated as a database-derived weight for prioritization and was not interpreted as binding affinity, receptor occupancy, dose, pharmacologic potency, or in vivo effect size. The antipsychotic drug set was anchored to the WHO Anatomical Therapeutic Chemical (ATC) N05A class, including typical and atypical antipsychotics and lithium under N05AN. Lithium was retained in the computational output because it is included in that ATC grouping, but it was interpreted cautiously because it is not a conventional antipsychotic.

The TWAS input consisted of ancestry-specific type 2 diabetes results derived from the Suzuki et al. multi-ancestry GWAS [10]. Five ancestry directories were analyzed: African American or admixed African ancestry labeled AFA, East Asian ancestry labeled EAS, European ancestry labeled EUR, Hispanic or admixed American ancestry labeled HIS, and South Asian ancestry labeled SAS. Each directory included six tissue-level S-PrediXcan result files: adipose subcutaneous, adipose visceral omentum, liver, skeletal muscle, pancreas, and whole blood. These tissues were chosen because they are relevant to insulin sensitivity, adiposity, hepatic metabolism, endocrine pancreas function, glucose uptake, systemic inflammation, and accessible blood expression signatures.

For each tissue, TWAS genes were standardized by gene symbol, z-scores were converted to numeric values, and duplicate genes were resolved by retaining the row with the largest absolute z-score. The primary threshold for a significant diabetes-associated TWAS gene was an absolute z-score of at least 3. This threshold corresponds to an approximate two-sided p-value of 0.0027 and was selected as a pragmatic screening threshold to restrict the primary analysis to relatively strong gene-level associations while preserving sufficient signals for cross-tissue comparison. It was not treated as a transcriptome-wide significance threshold; robustness was examined at absolute z-score thresholds of 4 and 5. For each drug in each tissue, the analysis intersected the drug’s target genes with modeled TWAS genes. The core risk score was calculated as the sum, across significant matched target genes, of the absolute TWAS z-score multiplied by the DGIdb interaction score. This weighting was intended to prioritize overlap at targets with stronger diabetes-associated expression signals and higher database-assigned interaction weights. It was designed as an ordinal prioritization statistic rather than a validated pharmacologic or clinical risk model. Higher values therefore indicate stronger weighted overlap between a drug’s target genes and diabetes-associated genetically predicted expression. The score is best understood as a prioritization measure, not as a direct estimate of diabetes incidence. To avoid reliance on the weighted score alone, it was interpreted alongside unweighted target burden, hypergeometric enrichment, and threshold sensitivity analyses.

A hypergeometric enrichment test was also performed in each tissue to evaluate whether a drug’s modeled targets were over-represented among significant diabetes-associated TWAS genes relative to all modeled genes. Per-tissue enrichment p-values were corrected using the Benjamini-Hochberg false discovery rate procedure. Across tissues, enrichment p-values were combined using Stouffer’s method, followed again by false discovery rate correction. Cross-tissue aggregation summarized the number of tissues analyzed, mean and maximum risk score, total number of significant hits, mean number of significant hits, mean absolute z-score, and combined enrichment p-value and false discovery rate. A combined false discovery rate (FDR) below 0.05 was treated as formal evidence of target-set enrichment. Rankings or risk scores without combined FDR below 0.05 were retained as exploratory prioritization observations.

Follow-up analyses added several layers. First, drugs were prioritized if they passed a combined enrichment false discovery rate below 0.05, had a high absolute burden, or belonged to a known-biology list that included clozapine, olanzapine, haloperidol decanoate, haloperidol, quetiapine, and risperidone. The high-burden and known-biology criteria were exploratory follow-up rules and were not equivalent to statistically significant enrichment. Second, a stricter analysis retained hits with an absolute z-score of at least 4 and required significance in at least two tissues for a given drug-gene pair. Third, sensitivity analyses repeated scoring across z-score thresholds of 3, 4, and 5. Fourth, a curated metabolic-axis analysis examined GLP1R, GIPR, PPARG, SLC2A4, INSR, IRS1, PPARGC1A, ADIPOQ, TCF7L2, KCNJ11, and ABCC8. Finally, a directionality analysis separated target hits into genes upregulated in diabetes TWAS and genes downregulated in diabetes TWAS. A parallel option computed aggravating scores from upregulated diabetes hits and compensatory scores from downregulated diabetes hits. Positive and negative TWAS z-scores were operationally described as upregulated and downregulated diabetes-associated expression signals; these labels do not measure expression changes in exposed patients or establish whether a drug increases or decreases gene expression.

The directionality analysis was deliberately simple and should be interpreted with caution. It used disease-associated expression direction only. It did not encode whether a drug is an agonist, antagonist, inverse agonist, inhibitor, or activator at the relevant target. This limitation is important for antipsychotics, many of which act through receptor blockade or inverse agonism. Therefore, “aggravating” and “compensatory” are provisional labels rather than definitive pharmacologic classifications. No external validation against patient-level metabolic outcomes was performed. Recovery of clinically recognized high-risk agents was used only as an internal face-validity check and does not validate the score as a predictor of diabetes incidence [1-6]. All analyses were performed in Python (Python Software Foundation, Wilmington, DE, USA) using pandas, numpy, scipy, and statsmodels. The study was hypothesis-generating and did not involve patient-level clinical outcomes.

## Results

Cross-ancestry patterns and overall burden

The amount of signal differed markedly across ancestry-specific TWAS directories. The European ancestry analysis produced the largest number of gene-hit rows, followed by East Asian, African ancestry, Hispanic, and South Asian analyses. In the follow-up outputs, EUR contained 320 gene-hit rows, EAS 158, AFA 82, HIS 56, and SAS 42. This pattern should be considered when interpreting cross-directory rankings. A larger and more polygenic signal in one ancestry directory may reflect sample size, GWAS power, TWAS model performance, linkage disequilibrium, allele frequency differences, or genuine heterogeneity in diabetes biology. The analysis therefore compared ancestry directories side by side rather than combining them into a formal cross-ancestry meta-analysis. Gene×tissue hit rows were not independent observations, and differences in directory-level totals were not tested as direct ancestry effects. The directory-level analysis inputs are summarized in Table 1.

**Table 1:** Directory-level analysis summary Data are presented as counts, N (%), and continuous TWAS association values as mean ± SD. Gene-hit row percentages are calculated using the total follow-up hit count across all directories (N = 658). TWAS association strength is represented by the Z-value. Gene-level screening uses an absolute Z-value of at least 3.0, corresponding to an approximate two-sided p-value of 0.0027. For enrichment analyses, a false discovery rate < 0.05 after Benjamini–Hochberg correction is considered statistically significant; rank order alone is exploratory. TWAS: transcriptome-wide association study; AFA: African American or admixed African; EAS: East Asian; EUR: European; HIS: Hispanic; SAS: South Asian

Ancestry directory	Tissues analyzed, N (%)	Drugs analyzed, N (%)	Gene-hit rows, N (%)	Overall |Z|, Mean±SD	Overall signed Z, Mean±SD	Primary threshold
AFA	6 (100.0%)	42 (100.0%)	82 (12.5%)	3.668±1.057	-1.012±3.701	|Z|≥3.0
EAS	6 (100.0%)	42 (100.0%)	158 (24.0%)	5.420±1.408	-2.255±5.141	|Z|≥3.0
EUR	6 (100.0%)	42 (100.0%)	320 (48.6%)	5.287±2.174	1.254±5.584	|Z|≥3.0
HIS	6 (100.0%)	42 (100.0%)	56 (8.5%)	3.770±1.015	1.319±3.706	|Z|≥3.0
SAS	6 (100.0%)	42 (100.0%)	42 (6.4%)	4.368±1.171	0.816±4.498	|Z|≥3.0

The leading drugs differed across ancestry-specific outputs. In AFA, haloperidol decanoate ranked first by mean risk score, followed by clozapine and fluspirilene. In EAS, iloperidone ranked first, followed by clozapine, lithium, haloperidol decanoate, and fluspirilene. In EUR, iloperidone again ranked first, followed by risperidone, haloperidol decanoate, trifluoperazine, and lithium. In HIS, trifluoperazine ranked first by a large margin, followed by chlorpromazine and risperidone. In SAS, quetiapine fumarate ranked first, followed closely by clozapine and olanzapine. These rankings show that no single drug dominated every ancestry-specific risk score. The top five drugs by mean risk score in each directory are summarized in Table 2. With the exception of EUR trifluoperazine and EUR fluspirilene, these rank orders did not meet the combined enrichment FDR threshold and should be interpreted as exploratory weighted-overlap observations rather than evidence of drug-specific metabolic risk. Quetiapine fumarate ranked first in SAS but had five significant gene×tissue hits and a combined FDR of 1.00; this was, therefore, an exploratory result rather than an enrichment-positive finding.

**Table 2:** Cross-directory snapshot: top five drugs by mean risk score Risk score and absolute TWAS Z-value are presented as mean ± SD across tissues. Significant target burden is presented as the total number of significant gene × tissue hits (N), with the mean significant target count and mean percentage of matched targets shown as N (%). Enrichment test statistics are reported as mean χ² (df = 1), and cross-tissue evidence is reported as the Stouffer combined Z-value with the combined p-value and FDR. A combined FDR < 0.05 is considered statistically significant for target-set enrichment; rows with higher FDR values represent exploratory ranking observations. TWAS: transcriptome-wide association study; FDR: false discovery rate; AFA: African American or admixed African; EAS: East Asian; EUR: European; HIS: Hispanic; SAS: South Asian; χ²: chi-square

Directory	Rank	Drug	Risk score, Mean±SD	Total significant hits, N	Mean significant targets, N (%)	|Z|, Mean±SD	Mean χ²(df=1)	Combined Z	Combined p-value	Combined FDR
AFA	1	Haloperidol decanoate	2.012±4.153	8	1 (4.5%)	1.133±1.189	0.26	-3.288	9.99E-01	1.00E+00
AFA	2	Clozapine	0.296±0.441	7	1 (3.3%)	1.059±1.076	0.636	-6.794	1.00E+00	1.00E+00
AFA	3	Fluspirilene	0.260±0.435	5	1 (8.1%)	1.513±2.228	1.605	-8.705	1.00E+00	1.00E+00
AFA	4	Promazine	0.229±0.355	2	0 (6.1%)	1.197±0.000	0.112	-12.747	1.00E+00	1.00E+00
AFA	5	Methotrimeprazine	0.208±0.321	2	0 (6.1%)	1.219±0.000	0.112	-12.747	1.00E+00	1.00E+00
EAS	1	Iloperidone	6.480±10.039	2	0 (5.4%)	1.244±0.046	0.014	-13.13	1.00E+00	1.00E+00
EAS	2	Clozapine	4.163±2.913	20	3 (10.5%)	1.437±1.356	0.638	1.153	1.24E-01	1.00E+00
EAS	3	Lithium	3.017±4.674	2	0 (3.1%)	1.091±0.000	0.131	-13.494	1.00E+00	1.00E+00
EAS	4	Haloperidol decanoate	1.192±2.158	10	2 (5.7%)	1.217±1.432	0.051	-1.417	9.22E-01	1.00E+00
EAS	5	Fluspirilene	0.657±0.340	9	2 (15.2%)	1.789±1.392	0.473	1.25	1.06E-01	1.00E+00
EUR	1	Iloperidone	9.363±14.417	4	1 (9.6%)	1.477±1.049	0.002	-7.479	1.00E+00	1.00E+00
EUR	2	Risperidone	5.863±3.536	19	3 (14.3%)	1.506±0.692	0.094	-0.817	7.93E-01	1.00E+00
EUR	3	Haloperidol decanoate	5.129±3.464	25	4 (14.6%)	1.884±3.880	0.125	-0.65	7.42E-01	1.00E+00
EUR	4	Trifluoperazine	4.421±5.583	25	4 (33.4%)	2.723±1.888	4.555	3.159	7.92E-04	1.66E-02
EUR	5	Lithium	2.604±4.573	8	1 (10.7%)	1.692±3.641	0.038	-1.901	9.71E-01	1.00E+00
HIS	1	Trifluoperazine	4.626±7.247	4	1 (5.0%)	1.089±2.749	0.315	-9.322	1.00E+00	1.00E+00
HIS	2	Chlorpromazine	0.173±0.182	6	1 (7.8%)	1.094±1.111	0.732	-5.409	1.00E+00	1.00E+00
HIS	3	Risperidone	0.140±0.126	4	1 (3.4%)	1.070±0.359	0.054	-6.882	1.00E+00	1.00E+00
HIS	4	Promazine	0.126±0.209	3	0 (7.1%)	1.115±0.512	1.037	-12.337	1.00E+00	1.00E+00
HIS	5	Droperidol	0.122±0.192	2	0 (9.7%)	1.169±0.720	0.349	-12.48	1.00E+00	1.00E+00
SAS	1	Quetiapine fumarate	0.571±0.592	5	1 (8.0%)	1.102±0.909	0.857	-5.247	1.00E+00	1.00E+00
SAS	2	Clozapine	0.533±0.351	6	1 (3.1%)	0.990±0.426	0.079	-3.574	1.00E+00	1.00E+00
SAS	3	Olanzapine	0.530±0.519	11	2 (6.7%)	1.225±0.656	1.336	-1.362	9.13E-01	1.00E+00
SAS	4	Methotrimeprazine	0.178±0.276	2	0 (6.7%)	1.657±0.422	0.28	-12.53	1.00E+00	1.00E+00
SAS	5	Sertindole	0.178±0.276	2	0 (5.6%)	1.294±0.422	0.195	-12.619	1.00E+00	1.00E+00

At the same time, several drugs recurred across analyses. Clozapine was the most consistent clinically coherent exploratory signal. It appeared among higher-ranked drugs in all ancestry directories, showed meaningful gene-hit burden in EAS and EUR, and had an aggravating directionality profile in every priority-drug directionality snapshot. Olanzapine was also repeatedly captured, especially through metabolic-axis genes. Haloperidol decanoate showed strong baseline burden in AFA, EAS, and EUR, but its directionality was often mixed or compensatory. Trifluoperazine emerged as the clearest, least expected candidate, with significant enrichment in EUR and top ranking in HIS. Fluspirilene also passed enrichment in EUR, although its overall interpretation was less clinically direct. No clozapine, olanzapine, haloperidol decanoate, or quetiapine results met the combined enrichment FDR threshold; their rank-based observations should therefore be interpreted separately from the statistically significant EUR enrichment findings. The priority-drug directionality snapshot is summarized in Table 3.

**Table 3:** Directionality snapshot for priority drugs Directionality is represented as gene×tissue hits, N, with UP- and DOWN-regulated diabetes-associated hits shown as N (%). Net direction is based on the majority sign of the TWAS Z-value. UP and DOWN denote the sign of the TWAS association and do not represent measured expression changes caused by drug exposure or direct pharmacologic effects. TWAS: transcriptome-wide association study; AFA: African American or admixed African; EAS: East Asian; EUR: European; HIS: Hispanic; SAS: South Asian; DM: diabetes mellitus

Directory	Drug	Hits, N	UP in DM, N (%)	DOWN in DM, N (%)	Net direction
AFA	Haloperidol decanoate	8	5 (62.5%)	3 (37.5%)	UP
AFA	Olanzapine	8	1 (12.5%)	7 (87.5%)	DOWN
AFA	Clozapine	7	4 (57.1%)	3 (42.9%)	UP
AFA	Risperidone	3	1 (33.3%)	2 (66.7%)	DOWN
EAS	Clozapine	20	13 (65.0%)	7 (35.0%)	UP
EAS	Olanzapine	15	3 (20.0%)	12 (80.0%)	DOWN
EAS	Risperidone	11	3 (27.3%)	8 (72.7%)	DOWN
EAS	Haloperidol decanoate	10	1 (10.0%)	9 (90.0%)	DOWN
EUR	Clozapine	27	20 (74.1%)	7 (25.9%)	UP
EUR	Haloperidol decanoate	25	11 (44.0%)	14 (56.0%)	DOWN
EUR	Trifluoperazine	25	15 (60.0%)	10 (40.0%)	UP
EUR	Olanzapine	23	7 (30.4%)	16 (69.6%)	DOWN
EUR	Fluspirilene	22	12 (54.5%)	10 (45.5%)	UP
EUR	Risperidone	19	9 (47.4%)	10 (52.6%)	DOWN
HIS	Clozapine	6	4 (66.7%)	2 (33.3%)	UP
HIS	Haloperidol decanoate	6	4 (66.7%)	2 (33.3%)	UP
HIS	Olanzapine	5	2 (40.0%)	3 (60.0%)	DOWN
HIS	Risperidone	4	2 (50.0%)	2 (50.0%)	UP
SAS	Olanzapine	11	4 (36.4%)	7 (63.6%)	DOWN
SAS	Clozapine	6	4 (66.7%)	2 (33.3%)	UP
SAS	Haloperidol decanoate	5	3 (60.0%)	2 (40.0%)	UP

Clozapine: the most clinically coherent cross-ancestry exploratory signal

Clozapine showed the most consistent pattern across exploratory rank-based analyses. In AFA, it ranked second by mean risk score, with seven significant hits. In EAS, it ranked second, with 20 significant hits. In EUR, it ranked seventh but had the highest burden among several clinically relevant drugs, with 27 significant hits. In HIS, it ranked seventh, with six hits, and in SAS, it ranked second, with six hits. This recurrent appearance across all five ancestry directories is notable because clozapine is already one of the strongest clinical positive controls for antipsychotic metabolic risk. However, no clozapine result met the combined enrichment FDR < 0.05, and its cross-directory recurrence should therefore be viewed as an exploratory internal coherence observation rather than formal statistical enrichment evidence.

The directionality analysis strengthened this interpretation. Clozapine showed a net upregulated, or aggravating, profile in every priority-drug directionality snapshot: 57.1% up in AFA, 65.0% up in EAS, 74.1% up in EUR, 66.7% up in HIS, and 66.7% up in SAS. The aggravating-versus-compensatory analysis produced the same general impression. Clozapine had an aggravating score of 1.685 and an aggravating ratio of 18.90 in AFA, 15.418 and 1.61 in EAS, 9.914 and 7.39 in EUR, 0.537 and 12.07 in HIS, and 2.009 and 1.69 in SAS. These values do not imply that clozapine causes diabetes through a single pathway, but they do show that its targets repeatedly overlap with diabetes-associated expression signals in a direction that the current model flags as concerning. The direction labels remain TWAS association signs and do not establish that clozapine changes these genes in the same direction in patients.

Clozapine’s gene-level drivers varied by ancestry. In AFA, GLP1R was a prominent axis hit, downregulated in both subcutaneous and visceral adipose TWAS. HLA-B, DTNBP1, and AMD1 also contributed. In EAS, HLA-C, ITIH3, AMD1, HLA-B, AOC1, and LEP were among the leading drivers. In EUR, AOC1, GLP1R, CBX1, HLA-B, HLA-C, and ITIH3 were prominent. In HIS, clozapine again overlapped GLP1R, along with DRD4 and HLA-B. In SAS, HLA-B, PRKAB2, and HNMT were observed. The recurrence of immune-related HLA signals and the repeated GLP1R overlap nominate several intersecting candidate pathways rather than a single demonstrated receptor mechanism.

The strongest translational thread for clozapine was GLP1R. In AFA, GLP1R was downregulated in diabetes TWAS for clozapine in subcutaneous and visceral adipose tissue, with z-scores around −5.38. In EUR, GLP1R was also downregulated in adipose tissues with z-scores around −8.76. In HIS, GLP1R was again downregulated in adipose tissues, although with a smaller magnitude. This repeated incretin-axis overlap is biologically plausible because GLP-1 receptor signaling affects glucose-dependent insulin secretion, glucagon suppression, gastric emptying, and satiety [16-18]. It also connects directly to clinical intervention, since GLP-1 receptor agonists have been studied as treatments for antipsychotic-associated weight gain and metabolic dysfunction, including in clozapine- or olanzapine-treated patients [19-21]. The repeated GLP1R overlap does not establish that altered GLP1R expression mediates clozapine-associated diabetes or the response to GLP-1 receptor agonists.

Olanzapine: strong metabolic-axis overlap despite directionality complexity

Olanzapine showed a different but equally important exploratory target-overlap pattern. Its overall rank varied across ancestry directories, but its mechanistic-axis hits were among the most interpretable in the analysis. In EAS, olanzapine overlapped with GIPR, GLP1R, and PPARG. In EUR, it showed strong overlap with GIPR, GLP1R, and PPARG. In SAS, PPARG was the main axis hit. In AFA and HIS, GLP1R appeared as an axis signal. This convergence across the incretin and adipocyte regulatory axis is consistent with olanzapine’s established clinical metabolic liability. None of the olanzapine findings met the combined enrichment FDR threshold, so these observations indicate pathway proximity rather than statistically significant target-set enrichment or a demonstrated mechanism.

The EUR olanzapine result was especially informative. GLP1R was downregulated in both subcutaneous and visceral adipose tissue, GIPR was downregulated in whole blood, and PPARG was downregulated across multiple tissues, including adipose, liver, skeletal muscle, and pancreas, with one upregulated whole-blood PPARG hit. The EAS analysis also showed PPARG downregulation across several tissues and GIPR downregulation in visceral adipose tissue, with a GLP1R-upregulated pancreas signal. In SAS, olanzapine showed repeated PPARG downregulation across adipose, liver, skeletal muscle, pancreas, and visceral adipose tissue. These results place olanzapine near core glucose, incretin, and adipose biology.

However, olanzapine also illustrates the limitations of simple directionality scoring. In the priority-drug directionality summaries, olanzapine was classified as net downregulated in every ancestry directory: 12.5% up in AFA, 20.0% up in EAS, 30.4% up in EUR, 40.0% up in HIS, and 36.4% up in SAS. In the aggravating-versus-compensatory analysis, olanzapine was mixed or potentially protective in AFA, EAS, and EUR, with very low ratios in AFA and EUR. In SAS, however, it showed an aggravating score of 2.046 and an aggravating ratio of 1.80, placing it among the strongest concern drugs for that directory.

This apparent contradiction should not be read as evidence that olanzapine is metabolically protective. The current directionality model does not know whether a drug activates or blocks a target. For receptor-targeted drugs, this missing pharmacologic sign can reverse interpretation. A gene downregulated in diabetes may be compensatory if a drug activates the pathway, but potentially harmful if the drug further blocks residual signaling. Olanzapine is clinically high-risk, and the TWAS axis results are better interpreted as evidence of pathway proximity involving GLP1R, GIPR, PPARG, and adrenergic receptors, rather than as a simple up-versus-down causal statement.

Quetiapine: an exploratory South Asian signal

Quetiapine fumarate ranked first by mean risk score in SAS, but this result consisted of five significant gene×tissue hits and had a combined FDR of 1.00. Quetiapine was retained for follow-up because it is clinically relevant to antipsychotic metabolic monitoring and generally has intermediate average liability relative to clozapine and olanzapine [2-6]. However, this isolated SAS ranking was not enrichment-positive and cannot be interpreted as evidence of ancestry-specific clinical risk.

Trifluoperazine: a notable enriched candidate in EUR and HIS

Trifluoperazine was the most notable, less expected candidate. In the EUR analysis, it ranked fourth by mean risk score, had 25 significant target hits, and passed combined enrichment correction with a false discovery rate of 0.0166. In the same EUR directory, only trifluoperazine and fluspirilene passed the combined enrichment threshold below 0.05. Although the number of individually enriched tissues was zero in the output, Stouffer-combined cross-tissue enrichment identified trifluoperazine as statistically enriched. This means its targets were over-represented among diabetes-associated TWAS genes across tissues in the EUR analysis under the study’s enrichment model. This was one of only two combined-enrichment-positive drug findings in the full analysis; the other was fluspirilene in EUR. The enrichment-positive drugs are summarized in Table 4.

**Table 4:** Drugs with significant target enrichment for diabetes-associated TWAS genes Risk score is presented as mean ± SD across tissues. Significant targets are presented as N, and the mean number of significant targets is presented as N (% of matched targets). Enrichment test statistics are reported as mean χ² (df = 1), together with the cross-tissue Stouffer combined Z-value, combined p-value, and Benjamini–Hochberg false discovery rate (FDR). A combined FDR < 0.05 is considered statistically significant for target-set enrichment. TWAS: transcriptome-wide association study; FDR: false discovery rate; EUR: European; χ²: chi-square

Directory	Drug	Rank	Risk score, Mean±SD	Total significant hits, N	Mean significant targets, N (%)	Tissues enriched, N (%)	Mean χ²(df=1)	Combined Z	Combined p-value	Combined FDR
EUR	Trifluoperazine	4	4.421±5.583	25	4 (33.4%)	0 (0.0%)	4.555	3.159	7.92E-04	1.66E-02
EUR	Fluspirilene	12	0.588±0.431	22	4 (37.2%)	0 (0.0%)	3.189	3.456	2.74E-04	1.15E-02

Trifluoperazine also showed strong threshold robustness. In EUR, its risk score remained stable across stricter z-score thresholds, with scores of 26.5247 at the z-score threshold of 3, 26.0512 at 4, and 25.6839 at 5. This stability contrasts with signals that collapse when marginal hits are removed. Its strict recompute score in EUR was 25.8415, ranking second after iloperidone. Key EUR drivers included CAMK2B, GLP1R, EHMT2, CBX1, CALY, and ADRA1A. GLP1R was downregulated in both subcutaneous and visceral adipose tissue with z-scores around −8.76, while CAMK2B and CBX1 contributed strong upregulated signals.

The HIS analysis gave trifluoperazine a different kind of support. It ranked first by mean risk score, and its signal was driven mainly by CALY, which was strongly upregulated in visceral adipose and pancreas, and by GLP1R, which was downregulated in adipose tissues. In the HIS aggravating-versus-compensatory analysis, trifluoperazine had an aggravating score of 27.686 and an aggravating ratio of 385.04. This was the dominant signal in that ancestry directory. The number of unique driver genes was small, so caution is required, but the strength and consistency of the CALY-driven HIS signal make trifluoperazine a reasonable candidate for follow-up.

Trifluoperazine’s clinical metabolic risk is less well established than clozapine’s or olanzapine’s. For that reason, the present findings should not be used to change prescribing practice. They do, however, justify closer examination in observational datasets and pharmacovigilance systems, especially for EUR and HIS populations. Its overlap with GLP1R also connects it to the same incretin-axis thread observed for clozapine and olanzapine.

Haloperidol decanoate: high burden with a mixed or compensatory SLC2A4 signal

Haloperidol decanoate showed high baseline burden in several ancestry directories. It ranked first in AFA, fourth in EAS, third in EUR, and ninth in SAS. In EUR, it had 25 significant hits and a mean risk score of 5.1286. In AFA, it had the highest mean risk score, driven in part by TOR1A, HSPA4, ANPEP, DTNBP1, and AMD1. In EAS and EUR, SLC2A4 was a major driver. In SAS, SLC2A4 again appeared strongly, with downregulation in skeletal muscle and pancreas. None of the haloperidol decanoate rankings met combined enrichment FDR<0.05, so its high burden remains an exploratory signal.

The SLC2A4 signal is biologically important. SLC2A4 encodes GLUT4, the insulin-responsive glucose transporter central to glucose uptake in skeletal muscle and adipose tissue. GLUT4 trafficking and membrane translocation are core components of insulin action [22-24]. In this analysis, SLC2A4 showed very strong downregulation in the diabetes TWAS for haloperidol decanoate. In EUR skeletal muscle, the SLC2A4 z-score was approximately −18.50, and in the pancreas, approximately −12.84. In SAS, SLC2A4 was downregulated in skeletal muscle and pancreas with z-scores around −7.12 and −7.00. These are among the strongest axis-gene signals in the study.

Yet the interpretation again depends on directionality and pharmacologic action. Haloperidol decanoate often had a large compensatory component in the Option 2 analysis. In AFA, its aggravating score was 3.004, but its compensatory score was 9.070, yielding a ratio of 0.33. In EAS, the ratio was 0.01. In EUR, the aggravating score was 8.888, but the compensatory score was 21.884, yielding a ratio of 0.41. In SAS, the ratio was 0.75. These findings suggest that haloperidol decanoate overlaps strongly with diabetes-associated expression programs, but much of the overlap involves genes that are downregulated in diabetes. Clinically, haloperidol is generally viewed as having lower metabolic risk than clozapine or olanzapine, and this mixed or compensatory tilt is not inconsistent with that clinical pattern.

The safest interpretation is that haloperidol decanoate shows pathway proximity to glucose uptake biology, especially through SLC2A4, but not necessarily direct high metabolic liability. It should remain subject to standard metabolic monitoring, particularly in long-term treatment, but the current TWAS evidence does not place it in the same category as clozapine or olanzapine.

Mechanistic axis and directionality summary

Across the metabolic-axis analysis, GLP1R was the most recurrent multi-drug signal. It appeared for clozapine, olanzapine, and trifluoperazine in AFA, EUR, and HIS, and in EAS as a pancreas signal. GIPR appeared mainly for olanzapine in EAS and EUR. PPARG appeared strongly for olanzapine in EAS, EUR, and SAS. SLC2A4 appeared most clearly for haloperidol decanoate in EAS, EUR, and SAS. These genes provide a compact mechanistic map of the main findings: incretin signaling for clozapine, olanzapine, and trifluoperazine; adipocyte and insulin-sensitivity biology for olanzapine; and GLUT4-mediated glucose uptake for haloperidol decanoate. These observations identify candidate shared pathways and do not demonstrate gene-level mediation of antipsychotic metabolic effects. The mechanistic-axis gene hits are summarized in Table 5.

**Table 5:** Mechanistic-axis gene hits Rows show detailed metabolic-axis TWAS hits. TWAS association strength is represented by the signed Z-value, with p-value shown for each gene-drug-tissue association. Counts are shown as N where applicable in other tables; this table reports individual gene-level observations. These gene-drug-tissue overlaps are not independent clinical events and should not be interpreted as evidence of causal mediation or direct drug-induced expression change. TWAS: transcriptome-wide association study; AFA: African American or admixed African; EAS: East Asian; EUR: European; HIS: Hispanic; SAS: South Asian; DM: diabetes mellitus

Directory	Gene	Drug	TWAS Z statistic	Direction in DM	p-value	Interaction score	Tissue
AFA	GLP1R	Clozapine	-5.38	DOWN	7.26E-08	0.00648	Adipose subcutaneous
AFA	GLP1R	Olanzapine	-5.38	DOWN	7.26E-08	0.00828	Adipose subcutaneous
AFA	GLP1R	Trifluoperazine	-5.38	DOWN	7.26E-08	0.0105	Adipose subcutaneous
AFA	GLP1R	Clozapine	-5.38	DOWN	7.26E-08	0.00648	Adipose visceral omentum
AFA	GLP1R	Olanzapine	-5.38	DOWN	7.26E-08	0.00828	Adipose visceral omentum
AFA	GLP1R	Trifluoperazine	-5.38	DOWN	7.26E-08	0.0105	Adipose visceral omentum
EAS	GIPR	Olanzapine	-3.01	DOWN	2.57E-03	0.687	Adipose visceral omentum
EAS	GLP1R	Clozapine	3.77	UP	1.64E-04	0.00648	Pancreas
EAS	GLP1R	Olanzapine	3.77	UP	1.64E-04	0.00828	Pancreas
EAS	GLP1R	Trifluoperazine	3.77	UP	1.64E-04	0.0105	Pancreas
EAS	PPARG	Olanzapine	-4.65	DOWN	3.34E-06	0.011	Adipose subcutaneous
EAS	PPARG	Olanzapine	-4.65	DOWN	3.34E-06	0.011	Liver
EAS	PPARG	Olanzapine	-4.65	DOWN	3.34E-06	0.011	Muscle skeletal
EAS	PPARG	Olanzapine	-4.65	DOWN	3.34E-06	0.011	Pancreas
EAS	PPARG	Olanzapine	-4.48	DOWN	7.30E-06	0.011	Adipose visceral omentum
EAS	SLC2A4	Haloperidol decanoate	-7.32	DOWN	2.49E-13	0.0211	Muscle skeletal
EAS	SLC2A4	Haloperidol decanoate	-5.12	DOWN	3.06E-07	0.0211	Adipose subcutaneous
EAS	SLC2A4	Haloperidol decanoate	-4.88	DOWN	1.06E-06	0.0211	Pancreas
EAS	SLC2A4	Haloperidol decanoate	-4.08	DOWN	4.49E-05	0.0211	Adipose visceral omentum
EUR	GIPR	Olanzapine	-6.86	DOWN	6.89E-12	0.687	Whole blood
EUR	GLP1R	Clozapine	-8.76	DOWN	1.90E-18	0.00648	Adipose subcutaneous
EUR	GLP1R	Olanzapine	-8.76	DOWN	1.90E-18	0.00828	Adipose subcutaneous
EUR	GLP1R	Trifluoperazine	-8.76	DOWN	1.90E-18	0.0105	Adipose subcutaneous
EUR	GLP1R	Clozapine	-8.76	DOWN	1.90E-18	0.00648	Adipose visceral omentum
EUR	GLP1R	Olanzapine	-8.76	DOWN	1.90E-18	0.00828	Adipose visceral omentum
EUR	GLP1R	Trifluoperazine	-8.76	DOWN	1.90E-18	0.0105	Adipose visceral omentum
EUR	PPARG	Olanzapine	-5.85	DOWN	4.92E-09	0.011	Adipose subcutaneous
EUR	PPARG	Olanzapine	-5.85	DOWN	4.92E-09	0.011	Liver
EUR	PPARG	Olanzapine	-5.85	DOWN	4.92E-09	0.011	Muscle skeletal
EUR	PPARG	Olanzapine	-5.85	DOWN	4.92E-09	0.011	Pancreas
EUR	PPARG	Olanzapine	5.44	UP	5.26E-08	0.011	Whole blood
EUR	PPARG	Olanzapine	-4.85	DOWN	1.22E-06	0.011	Adipose visceral omentum
EUR	SLC2A4	Haloperidol decanoate	-18.5	DOWN	1.93E-76	0.0211	Muscle skeletal
EUR	SLC2A4	Haloperidol decanoate	-12.84	DOWN	9.40E-38	0.0211	Pancreas
EUR	SLC2A4	Haloperidol decanoate	3.53	UP	4.18E-04	0.0211	Adipose visceral omentum
EUR	SLC2A4	Haloperidol decanoate	-3.51	DOWN	4.53E-04	0.0211	Adipose subcutaneous
HIS	GLP1R	Clozapine	-3.43	DOWN	6.04E-04	0.00648	Adipose subcutaneous
HIS	GLP1R	Olanzapine	-3.43	DOWN	6.04E-04	0.00828	Adipose subcutaneous
HIS	GLP1R	Trifluoperazine	-3.43	DOWN	6.04E-04	0.0105	Adipose subcutaneous
HIS	GLP1R	Clozapine	-3.43	DOWN	6.04E-04	0.00648	Adipose visceral omentum
HIS	GLP1R	Olanzapine	-3.43	DOWN	6.04E-04	0.00828	Adipose visceral omentum
HIS	GLP1R	Trifluoperazine	-3.43	DOWN	6.04E-04	0.0105	Adipose visceral omentum
SAS	PPARG	Olanzapine	-3.39	DOWN	6.90E-04	0.011	Adipose subcutaneous
SAS	PPARG	Olanzapine	-3.39	DOWN	6.90E-04	0.011	Liver
SAS	PPARG	Olanzapine	-3.39	DOWN	6.90E-04	0.011	Muscle skeletal
SAS	PPARG	Olanzapine	-3.39	DOWN	6.90E-04	0.011	Pancreas
SAS	PPARG	Olanzapine	-3.34	DOWN	8.29E-04	0.011	Adipose visceral omentum
SAS	SLC2A4	Haloperidol decanoate	-7.12	DOWN	1.12E-12	0.0211	Muscle skeletal
SAS	SLC2A4	Haloperidol decanoate	-7	DOWN	2.58E-12	0.0211	Pancreas

The directionality analysis was useful but incomplete. Clozapine’s cross-ancestry aggravating pattern was consistent with its clinical risk. Trifluoperazine showed an aggravating pattern in HIS and an enriched signal in EUR. Olanzapine, however, was often classified as downregulated or compensatory despite being a known high-risk drug. Haloperidol decanoate was also frequently mixed or compensatory despite strong SLC2A4 overlap. These examples make clear that disease-expression direction alone is not enough. The next version of this framework should annotate each drug-target pair with pharmacologic mode of action and recompute a pharmacologic aggravation score based on both disease direction and drug action. The complete tabular results of the multi-ancestry TWAS-informed antipsychotic-diabetes analysis is available in Appendix A.

## Discussion

This analysis used ancestry-specific type 2 diabetes TWAS results to prioritize approved antipsychotics by overlap between their target genes and diabetes-associated genetically predicted expression. Its primary output is a set of statistically tested and exploratory target-overlap hypotheses, not an estimate of clinical diabetes risk. Only trifluoperazine and fluspirilene met formal combined FDR enrichment criteria, both in EUR. All other drug rankings, including recurring clozapine and olanzapine patterns, are exploratory. The strongest mechanistic thread was the incretin axis, especially GLP1R and, for olanzapine, GIPR. Haloperidol decanoate showed a different pattern: strong pathway overlap with SLC2A4 and glucose uptake biology, but with a directionality profile that was often mixed or compensatory.

The clozapine result is the clearest positive control. Clozapine is already known to carry high metabolic risk, and this analysis found recurrent cross-ancestry burden with a generally aggravating directionality profile. This does not prove that the specific TWAS genes mediate clozapine-induced diabetes. It does suggest that clozapine targets are repeatedly close to genetically regulated diabetes expression programs. In a clinical translational context, that is important. A computational screen that fails to recover clozapine would be difficult to interpret. A screen that recovers clozapine across ancestry directories, with GLP1R and immune-metabolic signals, has stronger face validity. Because clozapine did not meet the combined enrichment FDR <0.05 in any directory, this recurrence is an internal face-validity observation rather than statistical validation of the score or evidence of a causal pathway.

Olanzapine provides a more complicated but clinically relevant example. Its axis hits were among the most biologically interpretable in the analysis: GLP1R, GIPR, PPARG, ADRA2A, and ADRA1B. PPARG is central to adipocyte differentiation, lipid handling, and insulin sensitivity, and its biology has long been tied to metabolic disease and antidiabetic drug mechanisms [25-27]. GLP1R and GIPR are central to incretin biology, insulin secretion, glucagon regulation, gastrointestinal motility, and satiety signaling [16,17,28]. These findings fit well with olanzapine’s known clinical profile. The fact that simple TWAS directionality often labeled olanzapine as compensatory should be interpreted as a limitation of the model, not as a contradiction of clinical evidence. However, the olanzapine findings did not meet the combined enrichment FDR threshold and should be interpreted as exploratory pathway-proximity observations rather than confirmed metabolic mechanisms.

Quetiapine merits a similarly cautious interpretation. Quetiapine fumarate had the highest mean risk score in SAS, but this finding included five hits and had a combined FDR of 1.00. It neither demonstrates heightened ancestry-specific risk nor changes the clinical evidence that quetiapine generally has lower average metabolic liability than clozapine and olanzapine while still contributing to metabolic burden in some patients [2-6].

Mechanistic insights

**Figure 1:**
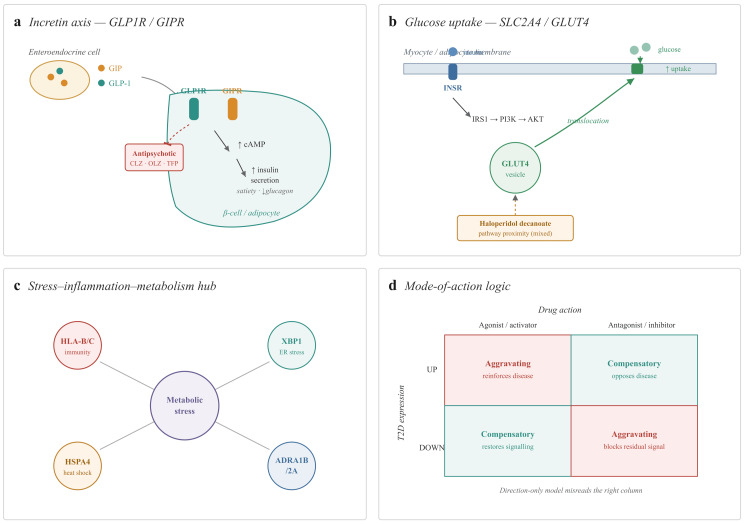
Candidate mechanistic axes linking antipsychotic target biology to type 2 diabetes pathophysiology Schematics are conceptual and hypothesis-generating; they depict candidate mechanistic axes, not study results. Flat-headed connectors denote inhibition or receptor blockade; arrowheads denote activation or downstream flow. This figure is a conceptual, hypothesis-generating synthesis of candidate pathway proximity. It is not an experimentally validated pathway model, and its arrows and inhibitory connectors do not establish the direction, magnitude, or tissue-specificity of drug effects in vivo. a) Incretin axis: Reduced incretin-receptor expression in T2D-associated programs (adipose/islet) coincides with antipsychotic targets. Receptor blockade may further blunt GLP-1/GIP tone affecting insulin secretion, glucagon suppression, and satiety, the rationale for GLP-1R-agonist countermeasures. b) Glucose uptake: GLUT4 (SLC2A4) mediates the rate-limiting step of insulin-stimulated glucose uptake in muscle and adipose. Its down-regulation is a core T2D feature; overlap with antipsychotic targets marks pathway proximity rather than confirmed high liability. c) Convergent hub: Immune, ER-stress, heat-shock, and adrenergic nodes converge on metabolic stress shared by schizophrenia and T2D. These signals are biologically plausible upstream modulators but less directly actionable than the incretin and GLUT4 axes. d) Mode-of-action logic: Disease direction alone is insufficient: true effect = expression direction × drug-action sign. Annotating each drug–gene pair (agonist/antagonist/inverse agonist) yields a signed pharmacologic aggravation score, resolving the apparent "compensatory" mislabelling of high-risk receptor blockers. CLZ: clozapine; OLZ: olanzapine; TFP: trifluoperazine; ER: endoplasmic reticulum; T2D: type 2 diabetes Image credit: Created by Ngo Cheung using Microsoft PowerPoint (Microsoft Corp., Redmond, WA, USA).

The incretin-axis result is the most promising translational signal (Figure 1). GLP1R appeared repeatedly across clozapine, olanzapine, and trifluoperazine, particularly in adipose tissues in AFA, EUR, and HIS. In Suzuki et al. [10], type 2 diabetes heterogeneity was linked to multiple biological processes, including pancreatic islet, adipocyte, endothelial, and enteroendocrine-related regulatory biology. The GLP-1 pathway sits naturally at the interface of glucose control, weight, gastrointestinal signaling, and brain-gut regulation. GLP-1 receptor agonists improve glycemic control and weight through multiple mechanisms, including glucose-dependent insulin secretion, reduced glucagon secretion, slower gastric emptying, and effects on appetite and body weight [17,18].

The potential clinical relevance is immediate because GLP-1 receptor agonists are already being used and studied as countermeasures for antipsychotic-associated weight gain. In a randomized clinical trial, liraglutide improved metabolic outcomes in patients treated with clozapine or olanzapine who had prediabetes and overweight or obesity [20]. Meta-analytic evidence also supports GLP-1 receptor agonists for antipsychotic-associated weight gain, although the evidence base remains smaller than in general obesity and diabetes care [19,21]. The TWAS overlap does not prove that GLP1R mediates the benefit of GLP-1 receptor agonists in antipsychotic-treated patients. It does provide a genetic-expression rationale for why this class of countermeasure is biologically coherent in the setting of clozapine and olanzapine exposure.

The GIPR result adds another layer. Olanzapine showed GIPR involvement in EAS and EUR. GIP and GLP-1 are both incretin hormones, and incretin biology is now central to diabetes and obesity therapeutics [16,28]. Dual incretin approaches may, therefore, be relevant to antipsychotic-treated patients with severe or early metabolic deterioration, although that remains a clinical question for future trials. The present analysis supports prioritizing incretin-related phenotyping in patients starting clozapine or olanzapine, including weight trajectory, appetite change, glycemic measures, and perhaps treatment response to GLP-1 receptor agonists. It does not establish a clinical role for dual-incretin therapy in antipsychotic-treated populations.

The SLC2A4 signal points to another pathway: insulin-stimulated glucose uptake. GLUT4 is central to skeletal muscle and adipose glucose transport, and impaired GLUT4 trafficking is a core feature of insulin resistance biology [22-24,29]. Haloperidol decanoate repeatedly overlapped SLC2A4 in EAS, EUR, and SAS, with particularly strong downregulated TWAS signals in skeletal muscle and pancreas. The interpretation is not that haloperidol is necessarily a strong diabetes-promoting drug. Rather, the drug’s target profile intersects a pathway that is central to diabetes pathophysiology. This distinction between pathway proximity and clinical liability is important. A drug can be near diabetes biology without being among the highest-risk agents in practice.

Immune and stress-response signals also recurred. Clozapine showed HLA-B and HLA-C overlap in EAS, EUR, HIS, and SAS. XBP1 contributed to fluspirilene and pimozide signals and is a core unfolded protein response factor. HSPA4 appeared for haloperidol decanoate, lithium, and chlorpromazine. These findings fit a broader view of type 2 diabetes as a disorder involving metabolic stress, inflammation, and endoplasmic reticulum stress, not only glucose and insulin pathways [30-35]. They may also be relevant to the high baseline inflammatory and cardiometabolic burden seen in schizophrenia and related disorders. However, these signals are less directly actionable than GLP1R, GIPR, PPARG, and SLC2A4.

Adrenergic targets appeared in several findings, including ADRA1B, ADRA1A, and ADRA2A. Adrenergic signaling influences lipolysis, hepatic glucose output, cardiovascular tone, and energy balance. Antipsychotic activity at adrenergic receptors may contribute to sedation, orthostasis, appetite, and metabolic outcomes. In this analysis, ADRA2A was seen with olanzapine, ADRA1B with olanzapine, promazine, methotrimeprazine, and sertindole, and ADRA1A with iloperidone, trifluoperazine, and ziprasidone. These signals are plausible, but their direction depends heavily on receptor pharmacology. They reinforce the need to move beyond gene-overlap scoring toward signed pharmacologic models.

Directionality and the pharmacologic mode-of-action gap

The directionality analysis was one of the most useful parts of the pipeline because it exposed a central limitation. Labeling upregulated diabetes genes as aggravating and downregulated diabetes genes as compensatory is reasonable as a first pass. It is not enough for antipsychotics. These drugs often block receptors. Some act as antagonists, inverse agonists, partial agonists, or functionally complex ligands depending on the receptor and cellular context. Therefore, the same TWAS direction can mean different things depending on drug action.

For example, if a gene is upregulated in diabetes and a drug activates that pathway, the drug might reinforce disease-associated biology. If the drug blocks that pathway, it could oppose the disease-associated state. Conversely, if a gene is downregulated in diabetes and a drug blocks residual signaling, the drug could worsen pathway insufficiency even though the simple model would call the hit compensatory. This is especially relevant for olanzapine, where many key axis hits were downregulated in diabetes, but the clinical drug is clearly high-risk. It is also relevant for haloperidol decanoate, where SLC2A4 is strongly downregulated, and the current output often appears compensatory.

The immediate methodological improvement is straightforward. Each drug-gene pair should be annotated with mode of action using resources such as ChEMBL, IUPHAR/BPS Guide to PHARMACOLOGY, DGIdb, and DrugBank [36-39]. The score should then be recomputed using both disease-expression direction and pharmacologic action. A signed pharmacologic aggravation score would not solve every problem, because receptor pharmacology can be tissue-specific and dose-dependent, but it would be more biologically valid than disease direction alone.

The second methodological improvement is robustness testing of interaction scores. Some high-ranking signals, especially iloperidone, were driven by very few genes with large interaction scores. In EAS and EUR, iloperidone ranked first by mean risk score and had very high aggravating scores, but the hit count was small and dominated by CNTF and ADRA1A. Sparse, high-score signals should not be interpreted in the same way as broad, cross-tissue, multi-gene burden. Capping interaction scores, rank-normalizing scores, rerunning unweighted overlap analyses, and excluding extreme single-gene drivers would clarify which findings are robust.

Study limitations

This study has several limitations. First, TWAS tests associations between genetically predicted expression and disease rather than observed expression in patients receiving antipsychotic treatment. Linkage disequilibrium, correlated expression-prediction models, and shared regulatory architecture can make noncausal genes appear associated with a trait [11,12]. Thus, a TWAS association may reflect a linked regulatory signal rather than the causal gene itself.

Second, tissue-specific prediction accuracy may differ by gene, tissue, ancestry dataset, linkage disequilibrium structure, allele frequency, GWAS sample size, and reference transcriptome performance. The six tissue-level models used here do not capture every relevant cell type, disease state, exposure condition, or developmental context. Accordingly, differences across AFA, EAS, EUR, HIS, and SAS directories may reflect biological heterogeneity, but they may also reflect differences in available data and model performance [10-12].

Third, the DGIdb-derived target lists and interaction scores are incomplete pharmacologic representations. They do not encode dose, treatment duration, formulation, plasma concentration, active metabolites, tissue distribution, receptor occupancy, molecular properties such as size and lipophilicity, polypharmacy, or the full functional context of receptor actions. They also do not fully distinguish agonism, antagonism, inverse agonism, partial agonism, and context-dependent signaling [13-15,36-39].

Fourth, the weighted risk score was not externally validated against diabetes incidence, hemoglobin A1c change, weight gain, or other clinical outcomes. The absolute Z-value threshold of 3 was a screening criterion rather than a transcriptome-wide multiple-testing threshold, and gene×tissue observations were not independent. Formal statistical inference in this study is therefore limited to the combined enrichment FDR analysis, in which only trifluoperazine and fluspirilene in EUR met FDR<0.05.

Finally, the study included no patient-level antipsychotic exposure data, clinical outcomes, medication adherence information, lifestyle variables, or longitudinal metabolic measurements. The findings therefore neither establish mechanisms of antipsychotic-induced metabolic toxicity nor support ancestry-specific prescribing or clinical decision-making.

Translational implications

The most immediate clinical implication is not to change prescribing based on this analysis but to refine the research agenda for precision monitoring. Clozapine and olanzapine already require intensive metabolic monitoring. The present findings support that practice and add mechanistic support for early attention to incretin and adipose pathways. Under existing guidelines, patients starting clozapine or olanzapine already warrant early weight trajectory monitoring, hemoglobin A1c or fasting glucose checks, and lipid monitoring; the present analysis does not provide a validated basis for changing those schedules or selecting a drug on the basis of TWAS results [1,7,8].

The findings also suggest that trifluoperazine deserves further clinical validation. In EUR, trifluoperazine passed combined enrichment correction with a false discovery rate of 0.0166 and had 25 significant target hits. In HIS, it ranked first and had a very high aggravating score driven mainly by CALY, with GLP1R involvement also present. These findings are not sufficient to classify trifluoperazine as a high metabolic-risk drug. They do support targeted pharmacovigilance and electronic health record (EHR)--based analyses, especially in EUR and HIS populations. A reasonable next study would compare incident diabetes, hemoglobin A1c trajectory, weight change, or hyperglycemia coding among patients exposed to trifluoperazine, clozapine, olanzapine, haloperidol decanoate, and lower-risk comparator drugs, with stratification by genetic ancestry.

Haloperidol decanoate illustrates how the framework may help distinguish pathway proximity from risk liability. Its SLC2A4 signal is strong, but the directionality profile is often mixed or compensatory. Clinically, haloperidol is not usually grouped with clozapine and olanzapine as a highest-risk metabolic agent. The TWAS result, therefore, suggests that a strong biological overlap does not automatically imply high clinical risk. It may instead identify pathways to monitor in certain contexts, such as long-term exposure, high dose, baseline insulin resistance, or ancestry-specific vulnerability.

Ancestry is central to this work. Suzuki et al. [10] emphasized that type 2 diabetes' genetic architecture and effect heterogeneity differ across ancestry groups, and that multi-ancestry analysis can improve biological understanding. In the present analysis, the strongest signals were not identical across AFA, EAS, EUR, HIS, and SAS directories. Clozapine was consistently concerning, but olanzapine, trifluoperazine, haloperidol decanoate, quetiapine, and iloperidone varied by ancestry directory. This does not mean that ancestry should be used simplistically or deterministically in prescribing. It does mean that validation studies should not default to European ancestry datasets and should not assume that risk models transfer without testing.

Large EHR and biobank networks could provide the next step. The All of Us Research Program, EHR-linked DNA biobanks, and OHDSI-style observational networks are well suited to evaluate medication exposure, ancestry, metabolic outcomes, and genetic risk at scale [40-42]. A practical validation study could test whether clozapine, olanzapine, trifluoperazine, and haloperidol decanoate differ in incident diabetes risk after adjustment for baseline metabolic status, diagnosis, co-medications, ancestry principal components, and social and clinical confounders. A stronger design would incorporate polygenic or partitioned type 2 diabetes scores, when available, and test drug-by-genetic-risk interactions.

Functional validation should proceed in parallel. Human adipocytes, hepatocytes, skeletal muscle cells, pancreatic beta-cell models, or induced pluripotent stem cell (iPSC)-derived systems could be exposed to clozapine, olanzapine, trifluoperazine, and haloperidol. Readouts should include GLP1R, GIPR, PPARG, SLC2A4, XBP1, and HSPA4 expression; GLP-1-stimulated cAMP signaling; insulin secretion; glucose uptake; and ER-stress markers. CRISPR interference or overexpression could test whether GLP1R or SLC2A4 modifies the metabolic response to antipsychotic exposure. These experiments would help determine whether the TWAS overlap reflects causal pathway involvement, compensatory changes, or simply shared genetic architecture.

The broader implication is that antipsychotic metabolic monitoring could eventually become more mechanism-informed. Current guidelines rightly emphasize universal monitoring because risk is common and serious. Only after prospective clinical and functional validation might future guidelines consider risk tiers informed by drug, baseline metabolic phenotype, ancestry-aware genetic risk, and pathway-level markers. The goal should not be to reduce monitoring in low-risk groups prematurely. It should be to identify patients who need earlier intervention, closer follow-up, or adjunctive therapy. The GLP1R signal is especially useful because it points to an existing therapeutic class rather than a distant drug-development target.

## Conclusions

This computational, hypothesis-generating TWAS-informed prioritization analysis mapped overlap between approved antipsychotic target genes and type 2 diabetes-associated genetically predicted expression across ancestry-specific datasets. It did not estimate clinical diabetes incidence, establish causal mechanisms, or validate an ancestry-specific prediction model. Clozapine and olanzapine were recurrent exploratory signals with biologically interpretable overlap involving GLP1R, GIPR, PPARG, and immune-metabolic pathways. Trifluoperazine and fluspirilene were the only drugs with combined enrichment FDR<0.05, both in EUR; trifluoperazine was additionally a leading exploratory signal in HIS. Haloperidol decanoate showed strong exploratory overlap with SLC2A4 and GLUT4 biology, but its directionality profile was mixed or compensatory and should not be interpreted as direct high metabolic liability.

The most important methodological next step is to add pharmacologic mode-of-action annotation to each drug-gene pair. Disease-expression direction alone is insufficient for receptor-targeted drugs. External EHR, biobank, and functional studies are needed to test whether the observed target-pathway overlaps predict metabolic outcomes or modify responses to interventions such as GLP-1 receptor agonists. If independently validated, this framework may help support more mechanism-informed metabolic monitoring and earlier adjunctive intervention.

## Appendices

Appendix A

Complete Tabular Results of the Multi-ancestry TWAS-Informed Antipsychotic-Diabetes Analysis

**Table 6:** Cross-directory snapshot: top five drugs by mean risk score FDR: false discovery rate; AFA: African American or admixed African; EAS: East Asian; EUR: European; HIS: Hispanic; SAS: South Asian

Directory	Rank	Drug	Mean risk score	|Z|, mean±SD	Significant targets, N (%)	Stouffer Z	Combined P	FDR
AFA								
AFA	1	Haloperidol decanoate	2.012	1.13±0.91	1 (4.5%)	−3.288	9.99e−01	1.00E+00
AFA	2	Clozapine	0.296	1.06±0.84	1 (3.3%)	−6.794	1.00E+00	1.00E+00
AFA	3	Fluspirilene	0.26	1.51±1.13	1 (8.1%)	−8.705	1.00E+00	1.00E+00
AFA	4	Promazine	0.229	1.20±1.02	0 (6.1%)	−12.747	1.00E+00	1.00E+00
AFA	5	Methotrimeprazine	0.208	1.22±0.97	0 (6.1%)	−12.747	1.00E+00	1.00E+00
EAS								
EAS	1	Iloperidone	6.48	1.24±0.82	0 (5.4%)	−13.130	1.00E+00	1.00E+00
EAS	2	Clozapine	4.163	1.44±1.52	3 (10.5%)	1.153	1.24e−01	1.00E+00
EAS	3	Lithium	3.017	1.09±0.75	0 (3.1%)	−13.494	1.00E+00	1.00E+00
EAS	4	Haloperidol decanoate	1.192	1.22±1.31	2 (5.7%)	−1.417	9.22e−01	1.00E+00
EAS	5	Fluspirilene	0.657	1.79±1.89	2 (15.2%)	1.25	1.06e−01	1.00E+00
EUR								
EUR	1	Iloperidone	9.363	1.48±1.34	1 (9.6%)	−7.479	1.00E+00	1.00E+00
EUR	2	Risperidone	5.863	1.51±1.32	3 (14.3%)	−0.817	7.93e−01	1.00E+00
EUR	3	Haloperidol decanoate	5.129	1.88±1.91	4 (14.6%)	−0.650	7.42e−01	1.00E+00
EUR	4	Trifluoperazine	4.421	2.72±2.50	4 (33.4%)	3.159	7.92e−04	1.66e−02
EUR	5	Lithium	2.604	1.69±1.58	1 (10.7%)	−1.901	9.71e−01	1.00E+00
HIS								
HIS	1	Trifluoperazine	4.626	1.09±1.15	1 (5.0%)	−9.322	1.00E+00	1.00E+00
HIS	2	Chlorpromazine	0.173	1.09±1.11	1 (7.8%)	−5.409	1.00E+00	1.00E+00
HIS	3	Risperidone	0.14	1.07±0.85	1 (3.4%)	−6.882	1.00E+00	1.00E+00
HIS	4	Promazine	0.126	1.11±1.04	0 (7.1%)	−12.337	1.00E+00	1.00E+00
HIS	5	Droperidol	0.122	1.17±0.78	0 (9.7%)	−12.480	1.00E+00	1.00E+00
SAS								
SAS	1	Quetiapine fumarate	0.571	1.10±1.02	1 (8.0%)	−5.247	1.00E+00	1.00E+00
SAS	2	Clozapine	0.533	0.99±0.78	1 (3.1%)	−3.574	1.00E+00	1.00E+00
SAS	3	Olanzapine	0.53	1.22±0.98	2 (6.7%)	−1.362	9.13e−01	1.00E+00
SAS	4	Methotrimeprazine	0.178	1.66±1.04	0 (6.7%)	−12.530	1.00E+00	1.00E+00
SAS	5	Sertindole	0.178	1.29±1.13	0 (5.6%)	−12.619	1.00E+00	1.00E+00

**Table 7:** Follow-up priority drugs selected by high absolute burden and/or known biology Priority 2 criteria were total significant hits ≥20 or inclusion on the prespecified known-biology list: olanzapine, clozapine, haloperidol decanoate, haloperidol, quetiapine, and risperidone. AFA: African American or admixed African; EAS: East Asian; EUR: European; HIS: Hispanic; SAS: South Asian

Directory	Primary rank	Drug	Risk score, mean±SD	Total significant hits, N	Mean |Z|, mean±SD
AFA	1	Haloperidol decanoate	2.012±4.153	8	1.133±1.189
AFA	2	Clozapine	0.296±0.441	7	1.059±1.077
AFA	7	Risperidone	0.190±0.452	3	0.997±1.040
AFA	13	Olanzapine	0.071±0.059	8	1.113±0.937
EAS	2	Clozapine	4.163±2.913	20	1.437±1.356
EAS	4	Haloperidol decanoate	1.192±2.158	10	1.217±1.432
EAS	6	Olanzapine	0.640±0.839	15	1.438±1.255
EAS	9	Risperidone	0.424±0.400	11	1.314±1.628
EUR	2	Risperidone	5.863±3.536	19	1.506±0.692
EUR	3	Haloperidol decanoate	5.129±3.464	25	1.884±3.880
EUR	4	Trifluoperazine	4.421±5.583	25	2.723±1.888
EUR	6	Olanzapine	2.149±2.664	23	1.678±1.538
EUR	7	Clozapine	1.876±2.506	27	1.806±1.986
EUR	12	Fluspirilene	0.588±0.431	22	2.625±1.968
HIS	3	Risperidone	0.140±0.126	4	1.070±0.359
HIS	7	Clozapine	0.097±0.150	6	1.007±0.388
HIS	10	Haloperidol decanoate	0.057±0.052	6	1.016±0.398
HIS	12	Olanzapine	0.051±0.080	5	1.113±0.381
SAS	2	Clozapine	0.533±0.351	6	0.990±0.426
SAS	3	Olanzapine	0.530±0.519	11	1.225±0.656
SAS	9	Haloperidol decanoate	0.087±0.111	5	1.013±1.849
SAS	36	Risperidone	0.000±0.000	0	0.896±NA

**Table 8:** Threshold-sensitivity analysis across |Z| cutoffs Each sensitivity cell is reported as risk score; number of significant gene×tissue hits; mean |Z|±SD. AFA: African American or admixed African; EAS: East Asian; EUR: European; HIS: Hispanic; SAS: South Asian

Directory	Drug	|Z|≥3.0: risk score; N hits; mean |Z|±SD	|Z|≥4.0: risk score; N hits; mean |Z|±SD	|Z|≥5.0: risk score; N hits; mean |Z|±SD
AFA				
AFA	Haloperidol decanoate	12.0740; 8; 3.9009±1.1890	9.0884; 2; 5.6163±1.2578	8.9374; 1; 6.5057±0.0000
AFA	Clozapine	1.7745; 7; 3.8328±1.0765	0.0698; 2; 5.3846±0.0000	0.0698; 2; 5.3846±0.0000
AFA	Fluspirilene	1.5614; 5; 4.0967±2.2279	0.9586; 1; 8.0799±0.0000	0.9586; 1; 8.0799±0.0000
AFA	Promazine	1.3761; 2; 4.3407±0.0000	1.3761; 2; 4.3407±0.0000	0.0000; 0; 0.0000
AFA	Methotrimeprazine	1.2450; 2; 4.3407±0.0000	1.2450; 2; 4.3407±0.0000	0.0000; 0; 0.0000
AFA	Perphenazine	1.1412; 3; 3.1293±0.1039	0.0000; 0; 0.0000	0.0000; 0; 0.0000
AFA	Risperidone	1.1405; 3; 3.6763±1.0397	1.0854; 1; 4.8754±0.0000	0.0000; 0; 0.0000
AFA	Chlorpromazine	1.0558; 7; 3.4119±0.5918	0.3102; 1; 4.7270±0.0000	0.0000; 0; 0.0000
AFA	Pimozide	1.0103; 5; 4.0967±2.2279	0.6203; 1; 8.0799±0.0000	0.6203; 1; 8.0799±0.0000
AFA	Lithium	0.9359; 2; 4.0470±0.9616	0.5465; 1; 4.7270±0.0000	0.0000; 0; 0.0000
AFA	Trifluoperazine	0.8074; 5; 4.0413±1.2297	0.1129; 2; 5.3846±0.0000	0.1129; 2; 5.3846±0.0000
AFA	Molindone	0.6154; 2; 3.0767±0.0706	0.0000; 0; 0.0000	0.0000; 0; 0.0000
AFA	Olanzapine	0.4229; 8; 4.0847±0.9372	0.3185; 4; 4.8626±0.6027	0.0891; 2; 5.3846±0.0000
AFA	Chlorprothixene	0.3692; 2; 3.0767±0.0706	0.0000; 0; 0.0000	0.0000; 0; 0.0000
AFA	Asenapine	0.3175; 4; 3.3068±0.2688	0.0000; 0; 0.0000	0.0000; 0; 0.0000
EAS				
EAS	Iloperidone	38.8783; 2; 3.9099±0.0462	0.0000; 0; 0.0000	0.0000; 0; 0.0000
EAS	Clozapine	24.9802; 20; 5.1367±1.3556	23.8745; 16; 5.5795±1.1233	8.9519; 9; 6.4565±0.5736
EAS	Lithium	18.1004; 2; 3.6406±0.0000	0.0000; 0; 0.0000	0.0000; 0; 0.0000
EAS	Haloperidol decanoate	7.1533; 10; 5.4934±1.4319	0.6607; 8; 5.9744±1.1306	0.4713; 6; 6.4725±0.7314
EAS	Fluspirilene	3.9422; 9; 5.9505±1.3919	3.9422; 9; 5.9505±1.3919	2.3303; 6; 6.6615±1.1060
EAS	Olanzapine	3.8396; 15; 4.8495±1.2553	0.5403; 11; 5.3989±0.9645	0.2084; 4; 6.5990±0.1457
EAS	Molindone	2.6398; 4; 6.5990±0.1457	2.6398; 4; 6.5990±0.1457	2.6398; 4; 6.5990±0.1457
EAS	Pimozide	2.6209; 12; 5.4028±1.5587	2.5765; 10; 5.7680±1.4336	1.5078; 6; 6.6615±1.1060
EAS	Risperidone	2.5460; 11; 4.8322±1.6284	1.1887; 6; 6.1217±0.9392	0.9115; 5; 6.4922±0.2701
EAS	Ziprasidone	2.3166; 5; 5.9225±1.5178	0.5109; 4; 6.5990±0.1457	0.5109; 4; 6.5990±0.1457
EAS	Quetiapine fumarate	1.9948; 5; 5.9225±1.5178	0.4400; 4; 6.5990±0.1457	0.4400; 4; 6.5990±0.1457
EAS	Chlorprothixene	1.5839; 4; 6.5990±0.1457	1.5839; 4; 6.5990±0.1457	1.5839; 4; 6.5990±0.1457
EAS	Amisulpride	1.3465; 1; 3.3530±0.0000	0.0000; 0; 0.0000	0.0000; 0; 0.0000
EAS	Trifluoperazine	1.2728; 9; 5.0046±1.5337	0.5570; 5; 6.1042±1.1135	0.5280; 4; 6.5990±0.1457
EAS	Loxapine	1.2184; 4; 6.5990±0.1457	1.2184; 4; 6.5990±0.1457	1.2184; 4; 6.5990±0.1457
EUR				
EUR	Iloperidone	56.1789; 4; 5.1578±1.0491	56.0858; 3; 5.6785±0.1549	56.0858; 3; 5.6785±0.1549
EUR	Risperidone	35.1782; 19; 4.1657±0.6925	22.0948; 11; 4.5452±0.6688	9.9764; 2; 5.7360±0.7580
EUR	Haloperidol decanoate	30.7717; 25; 5.3030±3.8797	22.3101; 14; 6.7013±4.7921	1.1245; 3; 15.2687±2.9157
EUR	Trifluoperazine	26.5247; 25; 5.8315±1.8878	26.0512; 21; 6.2847±1.7104	25.6839; 16; 6.9511±1.3790
EUR	Lithium	15.6266; 8; 5.5326±3.6415	15.1221; 6; 6.1658±4.0740	1.6718; 1; 14.4594±0.0000
EUR	Olanzapine	12.8938; 23; 5.0753±1.5380	5.5164; 17; 5.6017±1.4534	5.3012; 11; 6.3519±1.2549
EUR	Clozapine	11.2564; 27; 6.1410±1.9855	10.7487; 25; 6.3455±1.9162	10.0833; 18; 7.1622±1.6211
EUR	Ziprasidone	10.1871; 13; 4.5456±1.4817	0.4555; 8; 5.2051±1.5687	0.1399; 4; 6.3344±1.5274
EUR	Quetiapine fumarate	10.1607; 11; 3.7456±0.3810	2.0522; 5; 4.1201±0.1260	0.0000; 0; 0.0000
EUR	Aripiprazole lauroxil	9.6344; 6; 4.0080±0.2969	5.5751; 5; 4.1201±0.1260	0.0000; 0; 0.0000
EUR	Chlorpromazine	3.6682; 13; 7.2722±3.5923	3.3325; 11; 7.9608±3.4712	3.0681; 7; 10.1808±2.0655
EUR	Fluspirilene	3.5269; 22; 5.1547±1.9678	2.1959; 15; 5.9994±1.8334	1.6235; 9; 7.1869±1.3718
EUR	Perphenazine	3.0098; 13; 5.2301±1.6513	1.6660; 11; 5.5421±1.6038	0.3813; 5; 7.1399±0.7201
EUR	Pimozide	2.6883; 19; 5.7400±2.3989	1.8734; 14; 6.6408±2.1531	1.5493; 9; 8.0233±1.2228
EUR	Molindone	1.8630; 8; 4.9008±1.6729	1.8290; 7; 5.1223±1.6754	0.1985; 3; 6.5177±1.8160
HIS				
HIS	Trifluoperazine	27.7577; 4; 5.6924±2.7486	27.6858; 2; 7.9551±1.4779	27.6858; 2; 7.9551±1.4779
HIS	Chlorpromazine	1.0356; 6; 4.4153±1.1105	0.7769; 4; 5.0535±0.6526	0.7091; 3; 5.3605±0.2708
HIS	Risperidone	0.8387; 4; 3.4190±0.3592	0.0000; 0; 0.0000	0.0000; 0; 0.0000
HIS	Promazine	0.7580; 3; 3.6502±0.5115	0.2641; 1; 4.1325±0.0000	0.0000; 0; 0.0000
HIS	Droperidol	0.7331; 2; 3.6231±0.7204	0.4181; 1; 4.1325±0.0000	0.0000; 0; 0.0000
HIS	Methotrimeprazine	0.7146; 3; 3.6502±0.5115	0.1792; 1; 4.1325±0.0000	0.0000; 0; 0.0000
HIS	Clozapine	0.5814; 6; 3.4011±0.3875	0.0517; 1; 4.1325±0.0000	0.0000; 0; 0.0000
HIS	Lithium	0.4484; 2; 3.1103±0.0761	0.0000; 0; 0.0000	0.0000; 0; 0.0000
HIS	Sertindole	0.4004; 1; 3.7043±0.0000	0.0000; 0; 0.0000	0.0000; 0; 0.0000
HIS	Haloperidol decanoate	0.3395; 6; 3.3572±0.3980	0.0330; 1; 4.1325±0.0000	0.0000; 0; 0.0000
HIS	Loxapine	0.3384; 2; 3.6231±0.7204	0.1930; 1; 4.1325±0.0000	0.0000; 0; 0.0000
HIS	Olanzapine	0.3041; 5; 3.5619±0.3814	0.0990; 1; 4.1325±0.0000	0.0000; 0; 0.0000
HIS	Thioridazine	0.2005; 3; 3.6183±0.5095	0.0865; 1; 4.1325±0.0000	0.0000; 0; 0.0000
HIS	Asenapine	0.1759; 2; 3.6231±0.7204	0.1003; 1; 4.1325±0.0000	0.0000; 0; 0.0000
HIS	Pimozide	0.1710; 3; 3.6183±0.5095	0.0738; 1; 4.1325±0.0000	0.0000; 0; 0.0000
SAS				
SAS	Quetiapine fumarate	3.4235; 5; 3.9709±0.9093	0.2075; 2; 4.9374±0.4215	0.1100; 1; 5.2355±0.0000
SAS	Clozapine	3.1995; 6; 3.5062±0.4256	0.3033; 1; 4.2827±0.0000	0.0000; 0; 0.0000
SAS	Olanzapine	3.1819; 11; 3.6437±0.6559	0.1966; 2; 4.9374±0.4215	0.1042; 1; 5.2355±0.0000
SAS	Sertindole	1.0673; 2; 4.9374±0.4215	1.0673; 2; 4.9374±0.4215	0.5659; 1; 5.2355±0.0000
SAS	Methotrimeprazine	1.0673; 2; 4.9374±0.4215	1.0673; 2; 4.9374±0.4215	0.5659; 1; 5.2355±0.0000
SAS	Promazine	1.0105; 4; 5.4247±0.6136	1.0105; 4; 5.4247±0.6136	0.6410; 3; 5.6865±0.3918
SAS	Chlorpromazine	0.7685; 3; 5.8100±0.2935	0.7685; 3; 5.8100±0.2935	0.7685; 3; 5.8100±0.2935
SAS	Trifluoperazine	0.5912; 1; 3.3975±0.0000	0.0000; 0; 0.0000	0.0000; 0; 0.0000
SAS	Haloperidol decanoate	0.5220; 5; 5.0377±1.8491	0.2983; 2; 7.0570±0.0823	0.2983; 2; 7.0570±0.0823
SAS	Pimozide	0.1252; 2; 5.9121±0.0426	0.1252; 2; 5.9121±0.0426	0.1252; 2; 5.9121±0.0426
SAS	Asenapine	0.0766; 1; 3.1929±0.0000	0.0000; 0; 0.0000	0.0000; 0; 0.0000

**Table 9:** Maximum absolute TWAS Z statistic for curated metabolic-axis drug–gene overlaps TWAS: transcriptome-wide association study; AFA: African American or admixed African; EAS: East Asian; EUR: European; HIS: Hispanic; SAS: South Asian

Directory	Drug	Axis gene	Maximum |Z|	Direction at maximum signal
AFA	Clozapine	GLP1R	5.38	DOWN
AFA	Olanzapine	GLP1R	5.38	DOWN
AFA	Trifluoperazine	GLP1R	5.38	DOWN
EAS	Clozapine	GLP1R	3.77	UP
EAS	Haloperidol decanoate	SLC2A4	7.32	DOWN
EAS	Olanzapine	GIPR	3.01	DOWN
EAS	Olanzapine	GLP1R	3.77	UP
EAS	Olanzapine	PPARG	4.65	DOWN
EAS	Trifluoperazine	GLP1R	3.77	UP
EUR	Clozapine	GLP1R	8.76	DOWN
EUR	Haloperidol decanoate	SLC2A4	18.5	DOWN
EUR	Olanzapine	GIPR	6.86	DOWN
EUR	Olanzapine	GLP1R	8.76	DOWN
EUR	Olanzapine	PPARG	5.85	DOWN
EUR	Trifluoperazine	GLP1R	8.76	DOWN
HIS	Clozapine	GLP1R	3.43	DOWN
HIS	Olanzapine	GLP1R	3.43	DOWN
HIS	Trifluoperazine	GLP1R	3.43	DOWN
SAS	Haloperidol decanoate	SLC2A4	7.12	DOWN
SAS	Olanzapine	PPARG	3.39	DOWN

**Table 10:** Detailed curated metabolic-axis TWAS hits TWAS: transcriptome-wide association study; AFA: African American or admixed African; EAS: East Asian; EUR: European; HIS: Hispanic; SAS: South Asian

Directory	Gene	Drug	TWAS Z	Direction	p-value	Interaction score	Tissue
AFA							
AFA	GLP1R	Clozapine	−5.38	DOWN	7.26e−08	0.00648	Adipose subcutaneous
AFA	GLP1R	Olanzapine	−5.38	DOWN	7.26e−08	0.00828	Adipose subcutaneous
AFA	GLP1R	Trifluoperazine	−5.38	DOWN	7.26e−08	0.0105	Adipose subcutaneous
AFA	GLP1R	Clozapine	−5.38	DOWN	7.26e−08	0.00648	Adipose visceral omentum
AFA	GLP1R	Olanzapine	−5.38	DOWN	7.26e−08	0.00828	Adipose visceral omentum
AFA	GLP1R	Trifluoperazine	−5.38	DOWN	7.26e−08	0.0105	Adipose visceral omentum
EAS							
EAS	GIPR	Olanzapine	−3.01	DOWN	2.57e−03	0.687	Adipose visceral omentum
EAS	GLP1R	Clozapine	3.77	UP	1.64e−04	0.00648	Pancreas
EAS	GLP1R	Olanzapine	3.77	UP	1.64e−04	0.00828	Pancreas
EAS	GLP1R	Trifluoperazine	3.77	UP	1.64e−04	0.0105	Pancreas
EAS	PPARG	Olanzapine	−4.65	DOWN	3.34e−06	0.011	Adipose subcutaneous
EAS	PPARG	Olanzapine	−4.65	DOWN	3.34e−06	0.011	Liver
EAS	PPARG	Olanzapine	−4.65	DOWN	3.34e−06	0.011	Skeletal muscle
EAS	PPARG	Olanzapine	−4.65	DOWN	3.34e−06	0.011	Pancreas
EAS	PPARG	Olanzapine	−4.48	DOWN	7.30e−06	0.011	Adipose visceral omentum
EAS	SLC2A4	Haloperidol decanoate	−7.32	DOWN	2.49e−13	0.0211	Skeletal muscle
EAS	SLC2A4	Haloperidol decanoate	−5.12	DOWN	3.06e−07	0.0211	Adipose subcutaneous
EAS	SLC2A4	Haloperidol decanoate	−4.88	DOWN	1.06e−06	0.0211	Pancreas
EAS	SLC2A4	Haloperidol decanoate	−4.08	DOWN	4.49e−05	0.0211	Adipose visceral omentum
EUR							
EUR	GIPR	Olanzapine	−6.86	DOWN	6.89e−12	0.687	Whole blood
EUR	GLP1R	Clozapine	−8.76	DOWN	1.90e−18	0.00648	Adipose subcutaneous
EUR	GLP1R	Olanzapine	−8.76	DOWN	1.90e−18	0.00828	Adipose subcutaneous
EUR	GLP1R	Trifluoperazine	−8.76	DOWN	1.90e−18	0.0105	Adipose subcutaneous
EUR	GLP1R	Clozapine	−8.76	DOWN	1.90e−18	0.00648	Adipose visceral omentum
EUR	GLP1R	Olanzapine	−8.76	DOWN	1.90e−18	0.00828	Adipose visceral omentum
EUR	GLP1R	Trifluoperazine	−8.76	DOWN	1.90e−18	0.0105	Adipose visceral omentum
EUR	PPARG	Olanzapine	−5.85	DOWN	4.92e−09	0.011	Adipose subcutaneous
EUR	PPARG	Olanzapine	−5.85	DOWN	4.92e−09	0.011	Liver
EUR	PPARG	Olanzapine	−5.85	DOWN	4.92e−09	0.011	Skeletal muscle
EUR	PPARG	Olanzapine	−5.85	DOWN	4.92e−09	0.011	Pancreas
EUR	PPARG	Olanzapine	5.44	UP	5.26e−08	0.011	Whole blood
EUR	PPARG	Olanzapine	−4.85	DOWN	1.22e−06	0.011	Adipose visceral omentum
EUR	SLC2A4	Haloperidol decanoate	−18.50	DOWN	1.93e−76	0.0211	Skeletal muscle
EUR	SLC2A4	Haloperidol decanoate	−12.84	DOWN	9.40e−38	0.0211	Pancreas
EUR	SLC2A4	Haloperidol decanoate	3.53	UP	4.18e−04	0.0211	Adipose visceral omentum
EUR	SLC2A4	Haloperidol decanoate	−3.51	DOWN	4.53e−04	0.0211	Adipose subcutaneous
HIS							
HIS	GLP1R	Clozapine	−3.43	DOWN	6.04e−04	0.00648	Adipose subcutaneous
HIS	GLP1R	Olanzapine	−3.43	DOWN	6.04e−04	0.00828	Adipose subcutaneous
HIS	GLP1R	Trifluoperazine	−3.43	DOWN	6.04e−04	0.0105	Adipose subcutaneous
HIS	GLP1R	Clozapine	−3.43	DOWN	6.04e−04	0.00648	Adipose visceral omentum
HIS	GLP1R	Olanzapine	−3.43	DOWN	6.04e−04	0.00828	Adipose visceral omentum
HIS	GLP1R	Trifluoperazine	−3.43	DOWN	6.04e−04	0.0105	Adipose visceral omentum
SAS							
SAS	PPARG	Olanzapine	−3.39	DOWN	6.90e−04	0.011	Adipose subcutaneous
SAS	PPARG	Olanzapine	−3.39	DOWN	6.90e−04	0.011	Liver
SAS	PPARG	Olanzapine	−3.39	DOWN	6.90e−04	0.011	Skeletal muscle
SAS	PPARG	Olanzapine	−3.39	DOWN	6.90e−04	0.011	Pancreas
SAS	PPARG	Olanzapine	−3.34	DOWN	8.29e−04	0.011	Adipose visceral omentum
SAS	SLC2A4	Haloperidol decanoate	−7.12	DOWN	1.12e−12	0.0211	Skeletal muscle
SAS	SLC2A4	Haloperidol decanoate	−7.00	DOWN	2.58e−12	0.0211	Pancreas

**Table 11:** Cross-directory directionality snapshot for priority drugs AFA: African American or admixed African; EAS: East Asian; EUR: European; HIS: Hispanic; SAS: South Asian

Directory	Drug	Hits, N	UP in diabetes, N (%)	DOWN in diabetes, N (%)	Net direction
AFA	Haloperidol decanoate	8	5 (62.5%)	3 (37.5%)	UP
AFA	Olanzapine	8	1 (12.5%)	7 (87.5%)	DOWN
AFA	Clozapine	7	4 (57.1%)	3 (42.9%)	UP
AFA	Risperidone	3	1 (33.3%)	2 (66.7%)	DOWN
EAS	Clozapine	20	13 (65.0%)	7 (35.0%)	UP
EAS	Olanzapine	15	3 (20.0%)	12 (80.0%)	DOWN
EAS	Risperidone	11	3 (27.3%)	8 (72.7%)	DOWN
EAS	Haloperidol decanoate	10	1 (10.0%)	9 (90.0%)	DOWN
EUR	Clozapine	27	20 (74.1%)	7 (25.9%)	UP
EUR	Haloperidol decanoate	25	11 (44.0%)	14 (56.0%)	DOWN
EUR	Trifluoperazine	25	15 (60.0%)	10 (40.0%)	UP
EUR	Olanzapine	23	7 (30.4%)	16 (69.6%)	DOWN
EUR	Fluspirilene	22	12 (54.5%)	10 (45.5%)	UP
EUR	Risperidone	19	9 (47.4%)	10 (52.6%)	DOWN
HIS	Clozapine	6	4 (66.7%)	2 (33.3%)	UP
HIS	Haloperidol decanoate	6	4 (66.7%)	2 (33.3%)	UP
HIS	Olanzapine	5	2 (40.0%)	3 (60.0%)	DOWN
HIS	Risperidone	4	2 (50.0%)	2 (50.0%)	UP
SAS	Olanzapine	11	4 (36.4%)	7 (63.6%)	DOWN
SAS	Clozapine	6	4 (66.7%)	2 (33.3%)	UP
SAS	Haloperidol decanoate	5	3 (60.0%)	2 (40.0%)	UP

**Table 12:** Directionality output for all reported drugs, ranked by number of gene×tissue hits within directory Net direction is the majority sign of the TWAS Z statistic. It is a descriptive classification and does not encode agonism, antagonism, receptor occupancy, tissue-specific pharmacology, dose, formulation, or other drug-action characteristics. TWAS: transcriptome-wide association study; AFA: African American or admixed African; EAS: East Asian; EUR: European; HIS: Hispanic; SAS: South Asian

Directory	Drug	Hits, N	UP, N (%)	DOWN, N (%)	Net
AFA					
AFA	Haloperidol decanoate	8	5 (62.5%)	3 (37.5%)	UP
AFA	Olanzapine	8	1 (12.5%)	7 (87.5%)	DOWN
AFA	Chlorpromazine	7	2 (28.6%)	5 (71.4%)	DOWN
AFA	Clozapine	7	4 (57.1%)	3 (42.9%)	UP
AFA	Pimozide	5	2 (40.0%)	3 (60.0%)	DOWN
AFA	Trifluoperazine	5	2 (40.0%)	3 (60.0%)	DOWN
AFA	Fluspirilene	5	2 (40.0%)	3 (60.0%)	DOWN
AFA	Asenapine	4	1 (25.0%)	3 (75.0%)	DOWN
AFA	Risperidone	3	1 (33.3%)	2 (66.7%)	DOWN
AFA	Perphenazine	3	1 (33.3%)	2 (66.7%)	DOWN
AFA	Thioridazine	3	2 (66.7%)	1 (33.3%)	UP
AFA	Fluphenazine hydrochloride	2	1 (50.0%)	1 (50.0%)	UP
AFA	Chlorprothixene	2	1 (50.0%)	1 (50.0%)	UP
AFA	Aripiprazole lauroxil	2	1 (50.0%)	1 (50.0%)	UP
AFA	Methotrimeprazine	2	0 (0.0%)	2 (100.0%)	DOWN
EAS					
EAS	Clozapine	20	13 (65.0%)	7 (35.0%)	UP
EAS	Olanzapine	15	3 (20.0%)	12 (80.0%)	DOWN
EAS	Pimozide	12	2 (16.7%)	10 (83.3%)	DOWN
EAS	Risperidone	11	3 (27.3%)	8 (72.7%)	DOWN
EAS	Haloperidol decanoate	10	1 (10.0%)	9 (90.0%)	DOWN
EAS	Trifluoperazine	9	3 (33.3%)	6 (66.7%)	DOWN
EAS	Fluspirilene	9	2 (22.2%)	7 (77.8%)	DOWN
EAS	Perphenazine	8	2 (25.0%)	6 (75.0%)	DOWN
EAS	Chlorpromazine	8	2 (25.0%)	6 (75.0%)	DOWN
EAS	Thioridazine	7	1 (14.3%)	6 (85.7%)	DOWN
EAS	Asenapine	6	1 (16.7%)	5 (83.3%)	DOWN
EAS	Ziprasidone	5	1 (20.0%)	4 (80.0%)	DOWN
EAS	Quetiapine fumarate	5	1 (20.0%)	4 (80.0%)	DOWN
EAS	Fluphenazine hydrochloride	5	2 (40.0%)	3 (60.0%)	DOWN
EAS	Aripiprazole lauroxil	4	1 (25.0%)	3 (75.0%)	DOWN
EUR					
EUR	Clozapine	27	20 (74.1%)	7 (25.9%)	UP
EUR	Haloperidol decanoate	25	11 (44.0%)	14 (56.0%)	DOWN
EUR	Trifluoperazine	25	15 (60.0%)	10 (40.0%)	UP
EUR	Olanzapine	23	7 (30.4%)	16 (69.6%)	DOWN
EUR	Fluspirilene	22	12 (54.5%)	10 (45.5%)	UP
EUR	Risperidone	19	9 (47.4%)	10 (52.6%)	DOWN
EUR	Pimozide	19	13 (68.4%)	6 (31.6%)	UP
EUR	Thioridazine	18	13 (72.2%)	5 (27.8%)	UP
EUR	Fluphenazine hydrochloride	14	10 (71.4%)	4 (28.6%)	UP
EUR	Chlorpromazine	13	8 (61.5%)	5 (38.5%)	UP
EUR	Ziprasidone	13	7 (53.8%)	6 (46.2%)	UP
EUR	Perphenazine	13	12 (92.3%)	1 (7.7%)	UP
EUR	Quetiapine fumarate	11	6 (54.5%)	5 (45.5%)	UP
EUR	Prochlorperazine	11	8 (72.7%)	3 (27.3%)	UP
EUR	Asenapine	10	5 (50.0%)	5 (50.0%)	UP
HIS					
HIS	Chlorpromazine	6	3 (50.0%)	3 (50.0%)	UP
HIS	Clozapine	6	4 (66.7%)	2 (33.3%)	UP
HIS	Haloperidol decanoate	6	4 (66.7%)	2 (33.3%)	UP
HIS	Olanzapine	5	2 (40.0%)	3 (60.0%)	DOWN
HIS	Trifluoperazine	4	2 (50.0%)	2 (50.0%)	UP
HIS	Risperidone	4	2 (50.0%)	2 (50.0%)	UP
HIS	Thioridazine	3	3 (100.0%)	0 (0.0%)	UP
HIS	Promazine	3	2 (66.7%)	1 (33.3%)	UP
HIS	Pimozide	3	3 (100.0%)	0 (0.0%)	UP
HIS	Methotrimeprazine	3	2 (66.7%)	1 (33.3%)	UP
HIS	Asenapine	2	2 (100.0%)	0 (0.0%)	UP
HIS	Lithium	2	2 (100.0%)	0 (0.0%)	UP
HIS	Droperidol	2	2 (100.0%)	0 (0.0%)	UP
HIS	Loxapine	2	2 (100.0%)	0 (0.0%)	UP
HIS	Ziprasidone	2	2 (100.0%)	0 (0.0%)	UP
SAS					
SAS	Olanzapine	11	4 (36.4%)	7 (63.6%)	DOWN
SAS	Clozapine	6	4 (66.7%)	2 (33.3%)	UP
SAS	Haloperidol decanoate	5	3 (60.0%)	2 (40.0%)	UP
SAS	Quetiapine fumarate	5	2 (40.0%)	3 (60.0%)	DOWN
SAS	Promazine	4	4 (100.0%)	0 (0.0%)	UP
SAS	Chlorpromazine	3	0 (0.0%)	3 (100.0%)	DOWN
SAS	Pimozide	2	2 (100.0%)	0 (0.0%)	UP
SAS	Sertindole	2	2 (100.0%)	0 (0.0%)	UP
SAS	Methotrimeprazine	2	2 (100.0%)	0 (0.0%)	UP
SAS	Asenapine	1	0 (0.0%)	1 (100.0%)	DOWN
SAS	Trifluoperazine	1	1 (100.0%)	0 (0.0%)	UP

**Table 13:** Gene-level drivers reported for priority drugs in the follow-up analysis Detailed axis-gene Z statistics, p-values, interaction scores, and tissues are provided in Table 8. The driver-gene sets above reproduce the gene-level follow-up summaries for the priority drugs. AFA: African American or admixed African; EAS: East Asian; EUR: European; HIS: Hispanic; SAS: South Asian

Directory	Drug	Significant target genes, N	Gene×tissue hits, N	Reported driver genes
AFA	Clozapine	4	7	GLP1R; DTNBP1; HLA-B; AMD1
AFA	Haloperidol decanoate	5	8	TOR1A; HSPA4; ANPEP; DTNBP1; AMD1
AFA	Olanzapine	4	8	GLP1R; CHRM5; ADRA2A; AMD1
AFA	Risperidone	2	3	TNFRSF11A; AMD1
EAS	Clozapine	9	20	HLA-C; ITIH3; AMD1; HLA-B; AOC1; LEP
EAS	Haloperidol decanoate	4	10	SLC2A4; AMD1; HOMER1; EIF2AK4
EAS	Olanzapine	7	15	AMD1; ADRA2A; PPARG; GLP1R; HTR1E; EIF2AK4; GIPR
EAS	Risperidone	6	11	AMD1; UGT1A6; LEP; CYP1B1; ABCF1; EIF2AK4
EUR	Clozapine	9	27	AOC1; GLP1R; CBX1; HLA-B; ITIH3; HLA-C
EUR	Fluspirilene	7	22	XBP1; EHMT2; CBX1; HTT; AMD1
EUR	Haloperidol decanoate	13	25	SLC2A4; HSPA4; HOMER1; CAMK2G; TOR1A; ZIC4; ANPEP; ANKK1; AMD1
EUR	Olanzapine	9	23	GLP1R; GIPR; PPARG; ADRA1A; ADRA2A; AMD1
EUR	Risperidone	8	19	MSANTD1; UGT1A1; HTT; CYP1B1; LRP1B; AMD1; EIF2AK4
EUR	Trifluoperazine	8	25	CAMK2B; GLP1R; EHMT2; CBX1; CALY; ADRA1A
HIS	Clozapine	3	6	DRD4; GLP1R; HLA-B
HIS	Haloperidol decanoate	4	6	DRD4; ANPEP; HSPA4; MAPK14
HIS	Olanzapine	3	5	DRD4; ADRA1B; GLP1R
HIS	Risperidone	2	4	CYP1B1; UGT1A6
SAS	Clozapine	3	6	HLA-B; PRKAB2; HNMT
SAS	Haloperidol decanoate	2	5	SLC2A4; ANPEP
SAS	Olanzapine	4	11	ADRA1B; PPARG; PRKAB2; ADRA2A

Availability of data and materials

The data analyzed in this study were derived from publicly available resources and processed through the described TWAS-informed drug-gene interaction pipeline. The resulting summary outputs, including ancestry-stratified aggravating and compensatory drug scores, are available from the corresponding author upon reasonable request.

Clinical disclaimer

The findings are based on computational TWAS-informed drug-gene interaction analyses and should be interpreted as hypothesis-generating. They do not establish clinical safety, efficacy, or treatment recommendations for any antipsychotic drug. Further experimental and clinical validation is required. No medication selection, ancestry-based prescribing, or individual metabolic risk decision should be made on the basis of these results alone.
